# P-564. Ten-year Retrospective Review of Medical Records at Five Hospitals in the United States Highlights the Potential for Under-detection of Invasive Meningococcal Disease

**DOI:** 10.1093/ofid/ofaf695.779

**Published:** 2026-01-11

**Authors:** Julio A Ramirez, Stephen Furmanek, Thomas R Chandler, Josue Prado, Frederick Angulo, Lisa Harper, Steven Shen, Jessica Presa, Mohammad Ali, Raffaela Iantomasi, Jamie Findlow, Jennifer Moisi

**Affiliations:** Norton Healthcare, Louisville, Kentucky; Norton Healthcare, Louisville, Kentucky; Norton Healthcare, Louisville, Kentucky; Norton Healthcare, Louisville, Kentucky; Pfizer Vaccines, Portland, OR; Pfizer Vaccines, Portland, OR; Pfizer Canada ULC, Kirkland, Quebec, Canada; Pfizer, Inc., Collegeville, PA; Pfizer Inc., New York, New York; Pfizer Vaccines, Portland, OR; Pfizer Ltd, Tadworth, England, United Kingdom; Pfizer Vaccines, Portland, OR

## Abstract

**Background:**

Invasive meningococcal disease (IMD) burden estimates are based upon public health surveillance which relies on reporting of laboratory confirmed cases to public health authorities. IMD is confirmed by identification of *Neisseria meningitidis* in a specimen from a normally sterile site by bacterial culture or a polymerase chain reaction (PCR) test. Guidelines advise that blood and cerebrospinal fluid (CSF) be collected from patients with meningitis and tested by culture and PCR for *N. meningitidis*. Administration of intravenous (IV) antibiotics prior to specimen collection will reduce the sensitivity of bacterial culture. To assess IMD laboratory confirmation methods, we reviewed the records of patients hospitalized with signs and symptoms of meningitis.

Table

**Figure:**
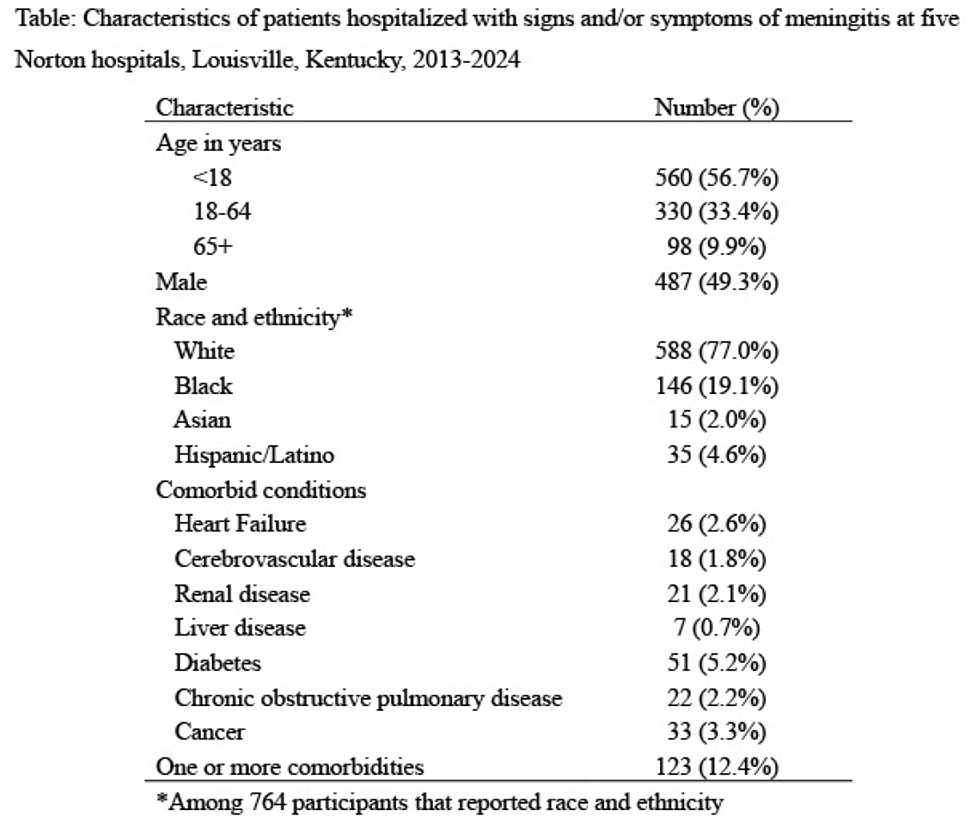

Figure 1

**Figure:**
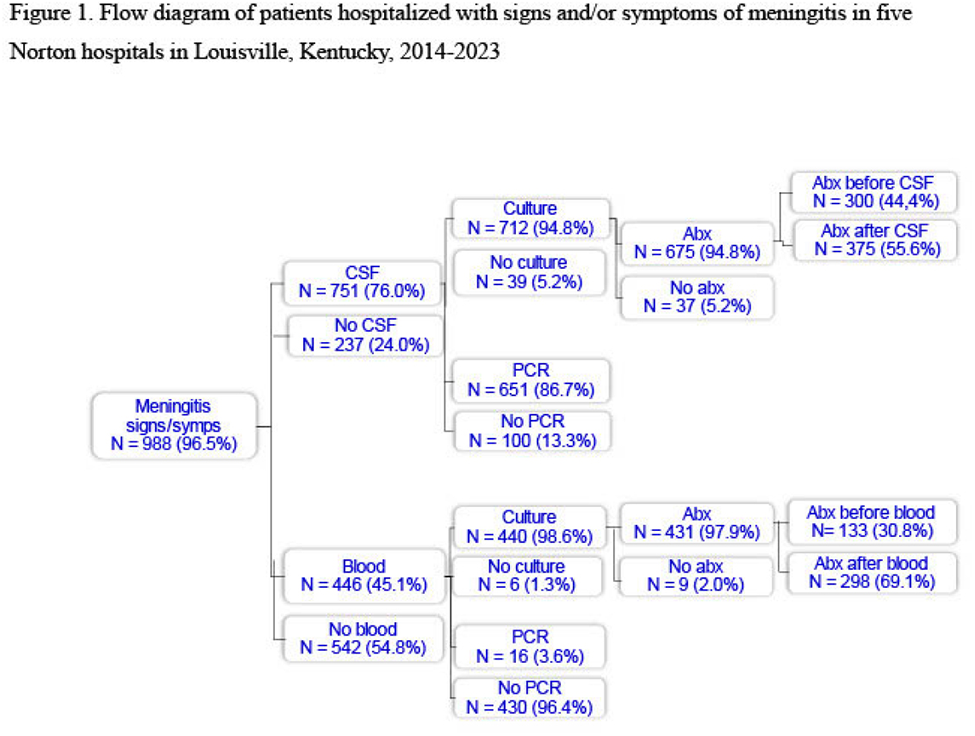

**Methods:**

Medical records were reviewed of patients hospitalized at the five Norton hospitals in Louisville, Kentucky in 2014-2023 with an ICD-10 or diagnosis-related group codes for meningitis or meningoencephalitis; only patients with recorded signs/symptoms of meningitis were included. The director of the clinical laboratory that received CSF and blood specimens was also interviewed to determine routine laboratory procedures during the study period.

Figure 2

**Figure:**
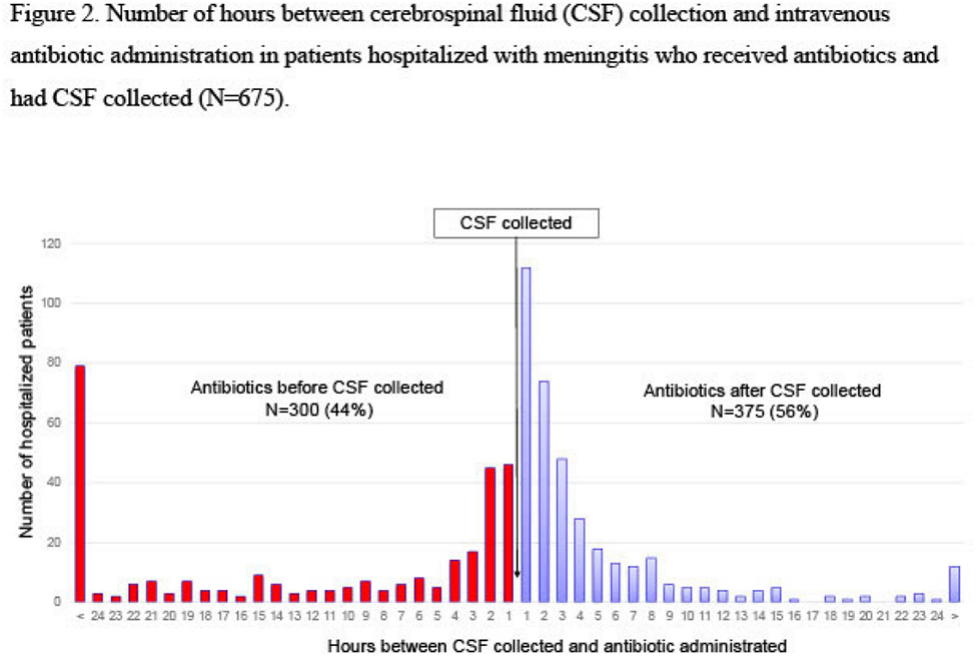

Figure 3

**Figure:**
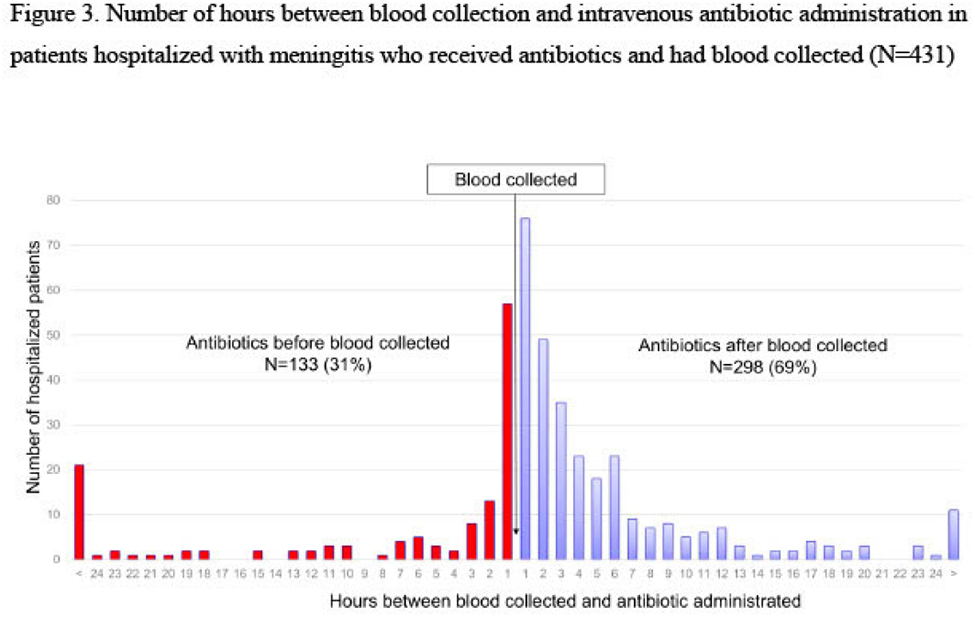

**Results:**

In 2014-2023, 988 patients were hospitalized at the Norton hospitals with signs and/or symptoms of meningitis (Table). Of the 988 patients, 751 (76%) had CSF collected and 446 (45%) had blood collected (Figure 1); 441 (45%) had both CSF and blood collected. Of collected CSF specimens, 712 (95%) were cultured and 651 (87%) were PCR-tested for *N. meningitidis*; 300 (44%) of cultured CSF specimens were collected after IV antibiotic administration (Figure 2). Of collected blood samples, 440 (99%) were cultured and 16 (4%) were PCR-tested for *N. meningitidis*; 133 (31%) of cultured blood specimens were collected after IV antibiotic administration (Figure 3).

**Conclusion:**

During a 10-year period in five Louisville hospitals, CSF and blood specimens were not routinely collected from hospitalized patients with meningitis. Furthermore, blood specimens from hospitalized patients with meningitis were not routinely PCR-tested for *N. meningitidis.* Finally, CSF and blood specimens were commonly collected after IV antibiotic administration.

**Disclosures:**

Julio A. Ramirez, MD, FACP, Pfizer Vaccines: Advisor/Consultant Frederick Angulo, DVM PhD, Pfizer Vaccines: Stocks/Bonds (Public Company) Lisa Harper, M.S., Pfizer Vaccines: Employer|Pfizer Vaccines: Stocks/Bonds (Public Company) Steven Shen, MD, PhD, Pfizer Canada ULC: Industry Jessica Presa, MD, Pfizer Inc: Industry|Pfizer Inc: Stocks/Bonds (Public Company) Mohammad Ali, PhD, Pfizer Vaccines: Employee|Pfizer Vaccines: Stocks/Bonds (Public Company) Raffaela Iantomasi, PhD, Pfizer Vaccines: Employer|Pfizer Vaccines: Stocks/Bonds (Public Company) Jamie Findlow, PhD, Pfizer Ltd: Industry|Pfizer Ltd: Stocks/Bonds (Public Company) Jennifer Moisi, PhD, Pfizer Vaccines: Employer|Pfizer Vaccines: Stocks/Bonds (Public Company)

